# To participate or not to participate: The troublesome question of nurses' conscientious objection to abortion: A qualitative study

**DOI:** 10.1111/jan.16258

**Published:** 2024-06-12

**Authors:** Valerie Fleming, Clare Maxwell, Claire Hanlon, Yvonne Robb, Joeri Vermeulen, Beata Dobrowolska

**Affiliations:** ^1^ Liverpool John Moores University Liverpool UK; ^2^ Glasgow Caledonian University (Retired) Glasgow UK; ^3^ Brussels Centre for Healthcare Innovation Brussels Belgium; ^4^ Vrije Universiteit Brussels Belgium; ^5^ Medical University of Lublin Lublin Poland

**Keywords:** abortion, conscience, human rights

## Abstract

**Aim:**

To report a study investigating the implementation of the “conscience clause” by practising nurses in two National Health Service Hospital Trusts in the UK.

**Design:**

A qualitative study.

**Methods:**

Data were collected from 2018 to 2020 through qualitative face‐to‐face interviews with 20 nurses, transcribed verbatim and analyzed by thematic analysis.

**Results:**

Major themes were developing conscience, negotiating conscience and parameters of participation.

**Conclusion:**

Participants had varied views on conscientious objection, reflecting a continuum from unwillingness to be near anything related to abortion to being willing to participate in the whole process. Most participants framed involvement as fulfilling their “duty of care” to their patient. Direct experience of witnessing abortion overrode faith‐based foundations to shape participants' beliefs as objectors or non‐objectors. Non‐objectors were supportive of objecting colleagues.

**Implications for the Profession:**

The complex nature of conscience as a fundamental human right is inherently related to the cultural and social context of nursing. “Employability” raised important questions over the real world of a nurse's legal right to invoke conscientious objection without consequences.

**Impact:**

Problem addressed Conscientious objection to abortion continues to affect nursing. Main findings There was little knowledge of the law and a reluctance to make formal objections. Where and on whom will the research have an impact It highlights the need for delineated and implemented guidelines on conscientious objection in practice for nurses. Its findings, while local, may be applicable to other abortion services.

**Patient and Public Contribution:**

Representatives of each were key in our advisory group.

**Reporting Method:**

COREQ checklist for qualitative research.

## 1 INTRODUCTION

The United Nations' (UN, 1948) founding document was its Universal Declaration on Human Rights. It protects individuals' consciences; Article 18 states that ‘everyone has the right to freedom of thought, conscience or religion…’. This right has subsequently been incorporated into other UN charters such as the International Covenant on Civil and Political Rights (ICCPR) (UN, 1966) and the Council of Europe's (1950) Convention on Human Rights. The protection of individuals' consciences was also acknowledged by legislators in most of the United Kingdom when the Abortion Act and amendment were enacted (United Kingdom Government, 1967, 1990). In the Abortion Act, applicable in Scotland, England and Wales, conscientious objection to abortion is incorporated, paragraph 4 stating that ‘Subject to subsection (2) of this section, no person shall be under any duty, whether by contract or by any statutory or other legal requirement, to participate in any treatment authorised by this Act to which he has a conscientious objection’.

It is beyond the scope of this paper to provide a detailed history of the development of the Abortion Act, as this has already been covered elsewhere (Davis & Davidson, 2006). However, it is worth noting that while two opposing medical camps had powerful voices, David Steel, who had introduced the Private Members' Bill, was also influenced by the volume of submissions coming from Roman Catholic organizations and individuals arguing against the Bill. Some of these organizations, accompanied by the Royal Colleges of Nursing, Midwives and Hospital Matrons, successfully lobbied for the inclusion of Section 4 of the Abortion Act known as the ‘conscience clause’ (Fleming, 2022). The conscience clause was a late amendment to the original Bill but one that ultimately smoothed the way for the enactment of the legislation (Reeves, 2016). The inclusion of this clause was to protect the rights of people, holding a legitimate objection to participation in abortion, who would otherwise be expected to work in the teams providing the service. The right is not absolute; the caveat is that, in cases of life‐threatening emergencies, objectors have to participate.

The enactment of the legislation has not ended controversy about abortion and relevant to nurses was a UK case in 1981 (RCN vs. Department of Health and Social Security) which requested clarity on the legality of nurses taking part in mid‐trimester abortions which were carried out by medical means. The medically induced abortions, which were a new development in medical science, used a different procedure from what is now common practice but induced labour and nurses were required to care for the women during their labours.

In its initial hearing, the Royal College of Nursing stressed the technical nature of what in the judges' views amounted to ‘performing the abortion’. This comprised administration of the drugs via an intra‐uterine catheter, which would be completed on ‘expulsion of the fetus’ but did not seem to include assisting with the delivery of the fetus itself. In an appeal to the House of Lords, a majority of three to two ruled that by extension the term ‘medical practitioners’ (as in the Act) should be extended to include nurses and midwives (House of Lords, 1981).

How the legislation has been implemented in practice, however, is a cause for some concern as some nurses in particular have suggested that it is impossible for them to object (Self et al., 2023). This article, therefore, reports on a study carried out to investigate the implementation of the above ‘conscience clause’ by nurses in practice in two National Health Service (NHS) Hospital Trusts in the UK.

## 2 BACKGROUND

In the mid‐1960s, when the Abortion Bill was being deliberated in the UK parliament, the ethics of abortion were the subject of bitter debate, in particular between two leading professors of midwifery, both of whom were obstetricians rather than midwives. Hugh Baird of Aberdeen was strongly in support of abortion on social grounds, where poverty and multiple children have brought misery to families and Iain Donald of Glasgow opposed it for all but a very few reasons (Baird, 1965; Davis & Davidson, 2006; Donald, 1966; Donald et al., 1958). Both of them were very powerful men with their voices being heard in major fora such as parliament while the nurses' and midwives', who were primarily women, voices were unheard. In current times conscientious objection has become the subject of ethical arguments instead of the subject of abortion, which has now become an integral part of NHS service provision. Rather than being based on clinical practice, however, these arguments have mainly been restricted to the theoretical disciplines of philosophy, ethics and discussions on legal cases (Clarke, 2017; Wicclair, 2019). A few research reports derived from the clinical practice of medicine, midwifery, nursing and pharmacy are available but these are very much in the minority (Curlin et al., 2007; Fleming et al., 2018; Maxwell et al., 2021; Lamb, Evans, Babanko, et al., 2019).

Nursing research in particular is limited with a systematic review of reasons as to why nurses object, revealing only 10 articles in which nurses' participation in abortion was discussed (Fleming et al., 2018). Conversely, the review showed that 136 articles discussed issues concerning medical practitioners. This article reported that, while it is popular belief that health professionals invoke conscientious objection to abortion because of religious beliefs, the majority of explanations found for objecting were due to moral reasons. After the publication of this article Ko et al. (2020) conducted a survey in Korea, the results of which showed that less than 30% of the 128 nurses who responded to the invitation to participate were aware of their legal right to object on conscience grounds to providing abortion‐related services. Similar results were found in a study carried out in Spain in which 421 nurses responded to an online questionnaire (Torres‐Flores et al., 2019). Just over 64% of respondents claimed to have poor knowledge of their rights to object to certain procedures including abortion and 10.5% would object to providing abortion‐related services. The authors of this study concluded that there was a strong correlation between religious beliefs and conscientious objection but failed to show this in the reported findings. A scoping review by Merner et al. (2024) included nurses and, although somewhat selective, 28 of their 68 studies included nurses. While conscientious objection was not the primary focus of their review, it formed a major part of their work and concluded that there needed to be a challenge to the dominant discourse of conscience being ‘associated solely with objection to abortion’ (p. 21).

Dobrowolska et al. (2020) compared the impact of legislation in the UK and Poland, both of which have conscience clauses concluding that in the UK, there are huge inconsistencies in the Act's interpretation, while in Poland, nurses are ‘generally left on the margin or treated analogically to the medical profession’ (p. 176). A qualitative study carried out with 21 nurses in Turkey, where there is no provision for or against conscientious objection by nurses, concluded that nurses can make a conscientious objection to abortion but should prioritize their patients' needs, thus not providing any clear guidance. Several studies by a team led by Canadian nurse, Christina Lamb, (Lamb, 2016, Lamb, Evans, Babanko, Wang, & Kirkwood, 2019, Lamb, Evans, Babenko‐Mould, Wing, & Kirkwood, 2019), investigated the phenomenon of conscientious objection to abortion by nurses. While most of these articles are theoretically based, they include one small empirical study of eight nurses whose views on conscience and conscientious objection were explored in some depth. The author concluded that because the concept of conscience lacked a concrete definition in nursing, it was difficult for practitioners to invoke a conscientious objection, but those who did so lacked support in the practice setting (Lamb, 2016a, 2016b). The overall picture, therefore, in the literature shows that nurses have limited knowledge of their legal rights and if they do wish to object, how to go about this.

By way of comparison, the picture is different concerning obstetricians, gynaecologists and other medical practitioners. De Zordo (2018) recorded that in a study conducted in Italy and Catalonia medical practitioners have the right to object and in Italy, they frequently do so whereas in Catalonia fewer objections are lodged. The author attributes this to how the hospitals organize their services, abortions are carried out in general gynaecological areas in Italy whereas in Catalonia they are in separate facilities. A similar issue was noted in the small qualitative study carried out in Norway where medical practitioners were able to be true to their consciences and object but felt complicit when they referred women to alternative providers (Nordberg et al., 2014). Another qualitative study of medical practitioners in three Latin American countries concluded that conscientious objection was not an isolated phenomenon but part of preventing women from access to legal services Objection, they stated, is a tool used to ensure their jurisdiction as doctors by non‐negotiation of their power remains dominant in the health services (Casas, 2009). This position is supported by an editorial in the European Journal of Contraception and Reproductive Health Care which makes two proposals for the way ahead in medicine: (1) Do not allow it and (2) Allow it with clearly defined parameters (Bitzer, 2016). The largest study concerning medical practitioners' views is a stratified, randomized survey of 1144 participants practising medicine in the United States of America (9). The study's results suggest that when service users request morally controversial clinical interventions, male physicians and those who are religious will be most likely to express personal objections and least likely to disclose information about the interventions or refer patients to more accommodating providers. In the meantime, they recommended that physicians and patients engage in ‘respectful dialogue to anticipate areas of moral disagreement and to negotiate acceptable accommodations before crises develop’ p. 598.

The situation for nurses thus remains divided and lacks clarity. With the exception of Dobrowolska et al. (2020), which did not include an empirical component, none of the above studies was based in the United Kingdom. It did, however, emphasize the scope of practice of nurses in the UK is wider than in Poland. Thus, in the UK, the law offers more provisions for objection therefore there are more procedures whose implementation may lead to nurses invoking conscientious objection rights.

## 3 METHODS

### 3.1 The study

#### 3.1.1 Aims

The present study seeks to address the deficit of UK‐based research by critically analysing the discourses of nurses in response to the questions ‘should nurses be allowed to object to providing abortion services on conscience grounds,’ and ‘what aspects of the abortion process should nurses be allowed to object to?’

The research team comprised a nurse, a midwife and two research assistants both of whom held qualifications in psychology. In order best to answer the research questions, we employed a qualitative design This ensured rich in‐depth data were available for analysis. participants in‐depth. The COREQ checklist (Tong et al., 2007) for qualitative research was used to ensure we had covered all the appropriate elements necessary for a rigorous study.

### 3.2 Methods

#### 3.2.1 Study setting and recruitment

Participants in this study were recruited from two hospitals, one in England and one in Scotland. The researchers were allowed to access each hospital to discuss the project with nursing managers and gatekeepers. Information sheets were left with each of them and potential participants were asked to contact the research team. Both hospitals also advertised times when a researcher would be in the hospital and able to discuss the project in person with potential participants. Further recruitment was carried out through snowballing.

#### 3.2.2 Inclusion/exclusion criteria

Participants were registered nurses in clinical practice. They had to have experience working in areas in which abortions were carried out but not necessarily practising there at the time of data collection.

#### 3.2.3 Data collection

Data were collected between 2018 and 2020 through qualitative face‐to‐face interviews by all four members of the research team and, with permission, were audio‐recorded. A few guiding questions had been prepared but after the formalities, we asked the initial question ‘What work do you do with women seeking termination of pregnancy’? This usually guided the questions to follow, although, at the end of each interview, we ensured that we had covered everything in the interview guide and gave all participants the opportunity to raise any issues which we had not covered. Interviews lasted between 35 and 95 min. We were also interested in exploring other data sources, such as hospital guidelines on conscience objection as well as those of professional and regulatory bodies. In neither hospital were any guidelines on conscientious objection to abortion known to the participants. Those of professional and regulatory bodies have been discussed in the findings' section.

#### 3.2.4 Data analysis

Data were transcribed verbatim by two members of the research team and inputted into NVivo version 12 for data management purposes. Analysis was then undertaken using Braun and Clarke's (2006) six‐step thematic analysis technique, two of the researchers coding and categorizing themes and ideas in order to identify significant patterns within the data collected (27). The six steps comprised data familiarization, initial coding, generating initial themes, reviewing themes, refining themes and finally naming such themes. We deliberately chose this framework for analysis rather than a later iteration as it has been tried and tested and appears to be more robust. From the analysis process, 26 codes were identified and four main themes then emerged from these codes for nurse participants. As this analysis was data‐driven, the concepts and phenomena highlighted were discovered using a reflexive and inductive process.

#### 3.2.5 Ethical considerations

The main ethical issue in this study was the right to anonymity. While this is important in all research, with the sensitivities concerning life and people's job prospects involved in this study it was particularly so. Thus the only person who had access to the full details of participants was the principal investigator. When participants were recruited by the researchers, consent forms were given to the principal researcher who initially allocated each a study number. At a later date, when all data had been processed and excerpts used she then allocated pseudonyms to replace the study numbers. The details were kept on a non‐networked computer. The rights to autonomy and confidentiality were also important and adhered to at all times. Another important ethical consideration in this study was to ensure that participant views on conscientious objection to abortion, not their perspectives, professional or otherwise, on the rights or wrongs of abortion were elicited. To ensure this, all researchers reinforced this before the interview commenced and reminded each participant of the study's purpose; namely to explore their views on and experiences of conscientious objection to abortion, not what they considered to be the rights or wrongs of abortion as a legal medical procedure.

Ethics approval was granted by the university (UREC 18/NAH032) and access by the individual hospitals and via the National Health Service's Integrated Research Application System (246528).

#### 3.2.6 Rigour and reflexivity

To ensure the trustworthiness of the qualitative data during analysis, each interview transcript was independently coded by two researchers. The researchers compared and discussed their findings on a regular basis with reference to the original qualitative data, establishing accuracy and transparency of the evolving codes and themes Thus, a multi‐level approach as outlined by Maxwell et al. (2020) was adhered to, allowing the researchers to gain a deeper understanding of the participants' perceptions and motivations; in turn increasing validity and rigour. To maintain a reflexive journey throughout, prior to commencing data collection all four members of the research team took part in a recorded discussion concerning our views about health professionals' right to make a conscientious objection. This was repeated at various times during the process of data collection and analysis. Finally, when we were deciding on final themes we returned to these transcripts to ensure that our views were not overriding those of the participants.

## 4 FINDINGS

### 4.1 Participants

Twenty nurses, of whom 19 were women and one a man participated. Six declared themselves to be conscientious objectors All had been in practice for over ten years. The major themes of developing conscience, negotiating conscience and parameters of participation.

### 4.2 Developing conscience

The theme ‘developing conscience’ encompasses professional and personal influences, such as the education, upbringing and religion experienced by the nurses that inform their beliefs surrounding abortion in the development of conscientious objection. It was evident that while religion has played some part in the development of formative views towards abortion and conscience for both objecting and non‐objecting nurses, many nurses chose not to object on the basis of their faith teachings;

‘I do have a religious belief which informs all of my life. However, I'm not a ‘pro‐lifer’.’ Nadine

Rather, ‘*direct experience’* of abortion within a professional capacity was reported as a strong determinant of personal views around abortion. For example, one nurse recounted the experience of a colleague whose personal views of abortion altered as a direct result of their involvement in abortion;

‘My friend was all for abortion, he was a theatre tech….then his wife became pregnant and he fainted at the scan – when he saw the baby, the shape. The very next day he had to assist in an abortion and he went to pieces. He said get me out of here – what have I been part of all these years…. that was an epiphany for him. He's a fantastic father….’ Nadia

### 4.3 Conflicts of conscience

Conflicts of conscience represent the intrapersonal challenges nurses may experience when working in abortion services. ‘*Duty of care*’ dominates this theme. A sense of professional responsibility that compelled the participants to prioritize the needs of the patient above their own was highlighted;

‘Because it's part of care, my duty is to do no harm and do good.’ Nadine

‘…if you come in to a job like this then you know what you should have to face and at the end of the day it should be about the patient….’ Naomi

Prioritizing patients' needs occurred even if this jarred with participants' own feelings towards involvement in abortion as some nurses described this as a key component of their professional duty;

‘I think it's part of my job to put my patient at ease as quite often they feel embarrassed or that they've made a mistake or that….’ Naomi

One nurse, however, conceded it would be difficult to engender compassion within the care nurses are expected to deliver;

‘I hope my communication would be professional, “It's okay Mrs Smith, there are four of us here now, we're getting that bleeding under control, the surgeon's on the way.” But, I'm not sure… I think I would find it hard to do the hand‐holding and brow mopping. But, of course, if she grabs my hand I would hold it.’ Natasha

Relatedly, some nurses described ‘*being without judgement*’ as an important aspect of working within this field to fulfil their compassionate care towards the patient;

‘Compassion, empathy. My role as a healthcare professional, as a human being. I think the ‘don't throw a stone analogy at people in glass houses’.’ Nerissa

The issue of ‘*Employability*’ was a divisive topic with some nurses asserting that objectors do not have a role in abortion services on the grounds that providing abortion services constitutes a large proportion of the role. In this respective, some felt forethought of the job demands should be given before deciding to take a role offering abortion care;

‘But if you take on that role, you must be aware that that is potentially so, would you not? Because I thought about it. I thought, “If I'm going to be working in a surgical ward, if this happened, what would happen?” Nerissa

The perceived incompatibility of being a conscientious objector nurse working within a service area in which abortions were regularly carried out such as gynaecological wards, led some to question why an objector would opt to work within such services. This was discussed in the context of the potential implications this may have on their career, particularly if the nurse was to declare their objection at interview;

‘I feel it could be used to select or deselect candidates.’ Nancy

### 4.4 Negotiating conscience

This theme of ‘negotiating conscience’ comprises interpersonal conflicts that a nurse may encounter when navigating conscientious objection within their clinical duties, including its management within the workplace. While naturally related to the theme of conflicts of conscience, it is distinct as the context of the codes comprising this theme represent conflicts on an organizational rather than individual level. Many of the participants reported having direct experience with colleagues who conscientiously object. However, irrespective of their own personal view of conscientious objection, it was broadly viewed as a *‘*right’ by participants. Fundamental to this perceived right was an understanding that an individual's moral and ethical beliefs are embedded within conscience;

‘Conscience is… I think, partly, it's your spiritual beliefs, partly it's your religious beliefs. For some people, it's fear, it's your personality. All of those things go into it. Really, if it's conscientious objection, who is anybody else to say, ‘Your conscience is wrong’? We do, don't we, if somebody is psychopathic or narcissistic, we'd say, ‘You've got no conscience.’ Do you say to people, ‘You've got too much conscience’? At the end of the day, you have to live with yourself.’ Nina

Some participants drew upon the life issue that surrounds the abortion debate, highlighting that it is this which makes conscientious objection to abortion distinct to other ethical debates within healthcare;

‘I don't think personally it's so much to do with religion or upbringing – just the actual idea and common sense factor is there's life and they should be allowed to withdraw from anything that's going to be detrimental to any person.’ Nancy

Despite reporting a ‘*lack of discussion*’ and the ‘*need for guidance*’ on the management of conscientious objection within the workplace, ‘*conscientious objection in practice*’ was reported as being accommodated either informally or formally by the participants and their colleagues. Several participants expressed empathy towards their colleagues who conscientiously object, particularly those who have personal experiences, such as baby loss, that may mean they are reluctant to participate in abortion. In such incidences, the nurses described an informal practice of discretely accommodating their colleague's objection out of respect and acknowledgement that they work together as a team;

‘I think, probably, a lot of conscientious objection goes on under the parameters from if you're working in an area with supportive individuals. As you put it, “Well, they watch your back.” Along with a whole host of procedures… If somebody didn't want to look after a stillbirth, other people would step in to do it unless it was absolutely essential.’ Nina

The impact of conscientious objection was broadly understood from the perspective of mitigating potential patient or staff harm. In the case of patients, concern was expressed over potential delays in treatment or that the experience of conscientious objection may pose as a barrier to abortion access;

‘…we've got lots of patients who had tried to approach their GP for a referral and they had been told no, they didn't agree with it. Or they'd actually had a consultation with their GP face to face and their GP had said, “No.” Nellie

In contrast, discussions of the ‘*impact of conscientious objection on colleagues’* was in the context of increasing colleague workload;

‘I don't mind it [conscientious objection] being lawful, that's fine, but then you should really be told that you can't have a job in that place if you object to what they do. They're passing it over to their other colleagues to do.’ Nadine

Conscientious objection was perceived as potentially problematic for smaller abortion service providers who may be unable to defer to non‐objecting staff.

‘I suppose if you were short staffed it would be really difficult or maybe if you had a manager who didn't understand where you were coming from.’ Natalie

Nevertheless, it was reported that effective management could minimize the potential for negative patient or staff effects. For example, one nurse reported how discrete management of conscience averted adverse patient effects;

‘So we instigated a bit of a self‐declaration form that the patients used to fill in just while they were filling their registration forms in when they came into the clinic just click are you here for contraception, are you here, whatever? So, we would actually self‐stream the patients to the right practitioner anyway and that would just fall into part and parcel of that.’ Nellie

An alternative view was offered by a participant who sometimes acted as shift leader and so had to allocate nurses to patients:

‘But it would be very beneficial to me, or whoever was organising the rota or the care, to know that certain members of staff, whilst they object to social abortion, if a lady is carrying a severely handicapped? child they're happy to take part in that case, I would want to know. It's a very pragmatic issue. That would allow me to know who is happy to do what in what situations and I can allocate them appropriately.’ Natasha

### 4.5 Parameters of participation

The theme ‘parameters of participation’ encompasses nurses' views pertaining to what participation in abortion involved and the perceived parameters of that participation. Participants conceptualized their ‘*understanding of participation’* in abortion in either narrow or broad terms. Consistent with a broad perspective of participation, some nurses described participation as involving every element that contributes to the abortion process;

‘I think possibly the whole process. I mean I can see admitting a woman but being actually involved in the pre‐med process and then the actual process in theatre and then the recovery process.’ Nevaeh

‘For me, being in the vicinity – in the room where it's happening, in an area where your help was required to do this – that as far as I'm concerned is participating in the procedure.’ Nancy

In contrast, a narrow understanding of what constitutes participation in abortion was conceptualized as being limited to specific acts that contribute to abortion, specifically the administration of the abortifacient;

‘Well, I think if you objected then the only thing you could really object to is giving the tablets. Anything else is patient care.’ Nadine

Interpretation of the *‘limitations’* to participation abortion broadly corresponded to the circumstances surrounding the woman's decision for choosing abortion. This gave rise to an individual threshold of what is perceived as an acceptable abortion by some nurses. Some nurses expressed a low tolerance for social abortions as these were perceived as a method of contraception;

‘I think there are lots of things to consider when a woman goes for an abortion, but I don't agree with it being used as contraception. Yeah mistakes can happen, but you learn from that and there are responsibilities to think of when you're sexually active.’ Naomi

In contrast, nurses expressed sympathy towards women undergoing abortions for foetal abnormalities and/or rape, which were often viewed as acceptable even among objecting nurses;

‘If it's for severe abnormalities and the mum and dad have thought, “We can't really do this,” especially some things where the child is going to die either just shortly before or shortly after birth, I can't see any objections to that. I really can't. I can't see how people could object to that. It's distressing the mums and the dads don't want to do what they're doing. To say, “I object to that,” I think that's totally unfeeling towards what people are going through.’ Nadine

‘The reality is that sometimes people are abused or raped or in violent relationships where they feel they have no other choice and things, you know?’ Naomi

Other participants fostered a holistic approach to the limitations of abortion, describing these within the parameters of the law;

‘I think the law is there. It's quite black and white. I think that would be my approach. I think that if the law allows you to do something then that would be okay.’ Nellie

## 5 DISCUSSION

The current study shows that participants had varied views on the subject of conscientious objection, reflecting a continuum from unwillingness to be near anything related to abortion to being willing to participate in all parts of the abortion process. Very few of their responses, however, focused on the law's applicability to them, rather, most participants provided their own views of abortion and the contributing factors underpinning their abortion beliefs. Nevertheless, at the core of each theme is the concept of conscience, which remains to the fore across this particular healthcare professional group and is discussed below in the context of each of the four themes developed.

While not an explicit aim of the present study, participants offered their views as to what underpinned their consciences. Religion was often discussed as being a prominent feature in the development of their formative views of abortion either through their upbringing and/or education. While a small number of participants discussed their objection as being faith based, for the majority religion was not a direct determinant of their decision to object. For many of the nurses, their direct experience of witnessing abortion first‐hand appeared to override these foundations to affirm their current beliefs either as an objector or a non‐objector. The latter group were totally supportive of colleagues who were objectors. This is an important finding as the focus of research within the field of conscientious objection to abortion often cites religion as a key determinant of conscience (Strickland, 2012; Nordberg et al., 2014; Torres‐Flores et al., 2019). Conversely, the study of Fleming et al. (2018) showed that religion was not the major factor in influencing participation in abortion. This narrative dominates and preoccupies the current debate surrounding conscientious objection to abortion within the literature when, as the present study highlights, the nature of conscious objection to abortion is inherently complex and multi‐faceted. Arguably, the cultural and social context in which the objection may occur is of significance in determining the reasons for conscientious objection to abortion (Ko et al., 2020). Indeed, Fleming et al. (2018), in their systematic review examining reasons midwives and nurses conscientiously object found a total of 116 narrow reasons for objection with 81 in support and 35 against conscientious objection. ‘Moral reasons’, such as ‘*conscience is an inner voice that needs listening to*’ comprised the majority of reasons for objection on the grounds of conscience, with other reasons being for practical, legal and religious reasons of conscience, again delineating that underpinnings of conscientious objection is varied. Pawlikowski (2014), however, was clear that justification of conscientious objection is fundamentally based on the protection of human life rather than religion.

As noted in the introduction, policy documents such as the European Convention on Human Rights, state that every person has the right to freedom of thought, conscience and religion (Council of Europe, 1950); although, as with the Universal Declaration on Human Rights (UN, 1948), this is not legally binding. Additionally, an individual's position can be subject to change throughout the journey of life. However, no participants mentioned this or indeed the Nursing and Midwifery Council's Code of Practice (2020), the latter of which is binding upon nurses practising in the UK. It contains a recently added clause specifically limiting nurses' and midwives' right to conscientious objection, stating ‘you [the nurse or midwife] must tell your colleagues, your manager and the person receiving care if you have a conscientious objection to a particular procedure and arrange for a suitably qualified colleague to take over responsibility for that person's care’ (standard 4.4) In most practice settings in the UK, this is difficult to put into practice due to staffing ratios. While not mentioned by participants, the issue of the impact of an individual nurse's conscientious objection on their ‘employability’ in the service was commented upon by the participants in this study, which raises important questions about the real‐world reality of a nurse's legal right to invoke conscientious objection confidentially without consequences to their career security and progression. Such a dilemma has also been manifested in other professional groups such as pharmacists (Maxwell et al., 2020) with a resolution satisfactory to all not yet reached.

In order to acknowledge the subject of conscience, the participants first had to define participation in the abortion process. Although the nurses have diverse viewpoints on participation, they agree that their individual understandings of participation guide their conscience in some way. As stated by Lamb, Evans, Babanko, Wang, & Kirkwood, 2019), nurses are moral agents who may become ethically opposed to certain procedures due to their own lived experiences. In this study, some nurses explain significant events pertaining to abortions which have lived with them over time and have, in turn, changed their viewpoints and beliefs in one way or another. Thus, direct experiences are instrumental to developing and negotiating conscience.

Most participants, including many who identified as objectors, framed their involvement in abortion as fulfilling their ‘duty of care’ to their patient. In this way, the patients' needs appear to override professionals' right to object alongside their own personal priorities and needs. Other studies have found that such ethical conflicts of conscience can give rise to moral distress among professionals and relatedly stress of conscience (Lamb, Evans, Babanko, Wang, & Kirkwood, 2019). However, contrary to concerns of negative patient impacts raised within the literature (Kane, 2009), the present findings indicate that nurses' professional obligation to the patient take precedence with active steps taken by nursing staff to protect patients' rights to an abortion. This stance was supported in recent study on service users' perspectives on conscientious objection to abortion carried out in the UK in which it was concluded that service users supported health professionals' rights to object (Self et al., 2023).

While it would be remiss to dismiss previous findings that have found nurses would conscientiously object if it conflicted with their religious or moral beliefs (Lamb, Evans, Babanko, Wang, & Kirkwood, 2019, Lamb, Evans, Babenko‐Mould, Wing, & Kirkwood, 2019), the present findings do raise questions about the wider implications of effective management of conscientious objection within the workplace for example, to negate the potential detrimental effects of moral distress and or/stress of conscience upon the wellbeing of nursing staff. However, a lack of discussion on conscientious objection and clear protocols on how to implement it within practice was expressed by the participants. This is consistent with previous findings which support the need for clear guidance on conscientious objection to abortion (Harries et al., 2014; Maxwell et al., 2020).

## 6 STRENGTHS & LIMITATIONS

This study has a number of key strengths. It adds to the literature of conscientious objection by giving voice to nurses' opinions surrounding the extent and limitations to conscientious objection to abortion, an opportunity rarely afforded to this profession. In this way, the findings are timely in light of the changing way in which abortion is performed and duties corresponding to the abortion process require greater involvement of other health professionals including nurses (Kane, 2009).

A further strength of this study is that it highlights the multifaceted nature of factors underpinning development of conscience, further evolving understanding beyond religion as being a prominent determinant. This is an important finding as moving the focal point of conscience beyond religion allows the complex and multi‐faceted nature of conscience to be understood. This has implications for policymakers in the development of abortion guidance.

Finally, the inclusion of participants who self‐identify both as conscientious objectors and non‐conscientious objectors to abortion offers a balanced view of the topic area. Abortion remains a divisive and controversial issue. Including the accounts of both sides of the debate highlights that consensus on such a controversial issue can be achieved through open discussion. For example, conscientious objection was unanimously perceived as a right, with many nurses expressing that it should be accommodated if practical and possible. Importantly, this highlights the need for clarity and explicit guidance in the area of conscience from both sides of the debate as while informal practices of accommodation did appear to work well among the participants included within the study, it may become problematic in other abortion services across the country and/or indeed internationally.

In spite of the strengths, this study is not without its limitations. In particular, while participant numbers are appropriate for a qualitative study, they were only recruited from two clinical areas both of which provided abortion services from early pregnancy to foeticide at 30 plus weeks' gestation. As such, the findings should be considered within the broader context of the type of abortion services where participants were recruited. Therefore, caution should be exercised when considering the transferability and generalizability of the findings to nurses who work in other services in which abortion plays a part.

While the limited literature available has shown similar issues concerning nursing in other parts of the world, this study has been restricted to the United Kingdom. It is hoped, however, that readers may be able to transfer findings to their own settings if they see a resonance. What would also be important in future studies is to investigate the impact of conscientious objection on service users.

## 7 CONCLUSION

This study explored the application of the ‘conscience clause’ among nurses practising within two National Health Service (NHS) Hospital Trusts in the UK regarding the development of conscience, negotiation of moral principles and the delineation of participation boundaries. Our results provide valuable insights into nurses' ethical decision‐making processes and their professional roles within healthcare contexts. The present study's findings highlight some of the complex nature of conscience underpinnings that are fundamentally a human rights' issue and are inherently related to cultural and social context. Thus, this study highlights the need for clearly delineated and implemented guidelines on conscious objection in practice for nurses and the need for nurses to be aware of those that do exist. Arguably, the professional obligation of nurses involved in this study to fulfil their patient's right to abortion above their own to conscientiously object works well within the services involved in this study. However, there remains potential for acrimony among colleagues who question the merits of objectors and their employability within services which offer abortion. Conscientious objection to abortion thus remains a contentious issue, with concerns over balancing the rights of the professional with those of the service user.

## AUTHOR CONTRIBUTIONS

Made substantial contributions to conception and design, or acquisition of data, or analysis and interpretation of data; VF, CM, CH. Involved in drafting the manuscript or revising it critically for important intellectual content; VF, CM, CH, YR, JV, BD. Given final approval of the version to be published. Each author should have participated sufficiently in the work to take public responsibility for appropriate portions of the content; VF, CM, CH, YR, JV, BD. Agreed to be accountable for all aspects of the work in ensuring that questions related to the accuracy or integrity of any part of the work are appropriately investigated and resolved; VF, CM, CH, YR, JV, BD.

## CONFLICT OF INTEREST STATEMENT

None of the authors declared nay conflict of interest in preparing this paper.

## FUNDING INFORMATION

We gratefully acknowledge the funding received from the Economic and Social Research Council to carry out this study (Grant number ES/R008841/1).

### PEER REVIEW

The peer review history for this article is available at https://www.webofscience.com/api/gateway/wos/peer‐review/10.1111/jan.16258.

## Data Availability

Data are available from https://opendata.ljmu.ac.uk/id/eprint/101/.
